# Wei-Mi-Shu Decoction ameliorates gastric mucosal injury in chronic gastritis with liver-stomach disharmony syndrome through the gut microbiota-hepatic bile acid metabolism axis

**DOI:** 10.3389/fphar.2026.1983808

**Published:** 2026-10-30

**Authors:** Xiaoyun Zou, Shouning Jia, Minmin Wei, Yongfu Qi, Hailing Li, Yan Liu, Junru Li

**Affiliations:** 1 Medical College, Qinghai University, Xining, China; 2 Department of Hepatopathy, Qinghai Provincial Hospital of Traditional Chinese Medicine, Xining, China; 3 Department of Research Institute of Traditional Chinese Medicine, Qinghai Provincial Hospital of Traditional Chinese Medicine, Xining, China; 4 Department of Cardiology, Qinghai Provincial Hospital of Traditional Chinese Medicine, Xining, China; 5 Department of Pharmacy, Qinghai Provincial Hospital of Traditional Chinese Medicine, Xining, China; 6 Department of Spleen and Stomach Diseases, Qinghai Provincial Hospital of Traditional Chinese Medicine, Xining, China

**Keywords:** bile acid metabolism, chronic gastritis, intestinal microbiota, Lgr5^+^ gastric stem cells, liver-stomach disharmony syndrome, Wei-Mi-Shu Decoction

## Abstract

**Introduction:**

Chronic gastritis (CG) is a high‐incidence disease in the digestive system, and liverstomach disharmony syndrome (LSD) is a relatively common syndrome type. This study aimed to evaluate the role and possible mechanism of traditional Chinese medicine Wei‐Mi‐Shu Decoction (WMS) in treating LSDCG.

**Methods:**

The CG and LSDCG animal models were established and treated with WMS to observe the TCM syndrome scores and behavioral changes, histopathological alterations of the gastric mucosa, and expressions of pro‐inflammatory cytokines and tight junction proteins. Then, the changes of gut microbiota and bile acids (BAs) were analyzed by microbiota sequencing and mass spectrometry, and the protein expressions of BA receptors were detected by immunohistochemistry and WB. Finally, Lgr5‐positive (Lgr5^+^) gastric cells were observed by immunofluorescence staining, the expression of gastric mucosal cell differentiation markers was detected by RT‐qPCR, and the fatty acid oxidation (FAO) of Lgr5^+^ cells was observed by fluorescence double staining.

**Results:**

WMS reduced the TCM syndrome scores, increased the autonomous activity ability, and improved gastric mucosal injury, inflammatory response, and gastric mucosal barrier disruption in the CG and LSDCG model rats. WMS also regulated the intestinal microbiota and BA metabolism of model rats, and promoted the levels of PPARα and RXR. In addition, WMS attenuated the loss of Lgr5^+^ gastric stem cells and promoted their differentiation and FAO.

**Conclusion:**

In conclusion, WMS promoted the differentiation of Lgr5^+^ gastric stem cells, potentially involving the gut microbiotahepatic BA metabolism axis to repair gastric mucosal injury in LSDCG.

## 1 Introduction

Chronic gastritis (CG) is a common and frequently-occurring gastrointestinal disease in clinical practice, accounting for over 90% of patients undergoing gastroscopy (Fang et al., 2018). It is characterized by persistent inflammation and injury of the gastric mucosa, often accompanied by upper abdominal pain, abdominal distension, and indigestion (Sipponen and Maaroos, 2015). Some patients may simultaneously present with gastroesophageal reflux and gastric dysfunction (Fink et al., 1984). CG is classified into chronic non-atrophic gastritis and chronic atrophic gastritis. Epidemiology shows that the incidence of CG increases with age and is positively correlated with the incidence of gastric cancer (Yin et al., 2022). At present, the treatment of CG in Western medicine mainly involves the use of antibiotics, proton pump inhibitors, and other drugs (Yang et al., 2024). Although these can alleviate the symptoms, they have many adverse reactions and a high recurrence rate after drug withdrawal (Rossi et al., 2023). In recent years, the advantages of traditional Chinese medicine (TCM) prescriptions in treating CG have gradually become prominent (Chen et al., 2023). Wei-Mi-Shu Decoction (WMS) is a long-term clinical practice prescription of Junru Li, a renowned chief physician of traditional Chinese medicine in Qinghai Province. It has been proven to have good clinical efficacy in treating chronic erosive gastritis, chronic atrophic gastritis, gastric ulcers and other diseases. And studies have confirmed that WMS can regulate immune responses, reduce inflammation, and enhance gastric mucosal repair (Guan et al., 2024). However, the specific mechanism by which it improves CG remains to be further explored.

In TCM theory, the imbalance of liver, spleen, and stomach functions (i.e., “liver-intestine-stomach axis”) is an important concept, which reflects the close connection among the liver, intestines, and stomach. The normal operation of this axis is crucial for maintaining overall health. If this balance is disrupted, it may trigger a series of health problems, including CG, especially the type of liver-stomach disharmony syndrome CG (LSDCG). Liver-stomach disharmony (LSD) syndrome is a prevalent pattern of CG in TCM, characterized by the imbalance between the liver and stomach (Liu et al., 2025). TCM believed that liver qi stagnation caused by factors such as emotional distress may further transform into internal heat, which in turn affects the digestive function of the stomach. When the spleen is weak and unable to transform food, it can also aggravate the symptoms of disharmony between the liver and stomach. Modern pharmacological studies have demonstrated that bile acid (BA) metabolism is an important physiological function of the liver (di Gregorio et al., 2021). Liver cells convert cholesterol into BAs and transport them to the intestine to participate in reactions. Subsequently, they are reabsorbed into the blood and return to the liver, forming the enterohepatic circulation of BAs(Chiang and Ferrell, 2018). Numerous studies have confirmed the importance of BA enterohepatic circulation in the onset and progression of chronic gastrointestinal diseases (Cai et al., 2022a). WMS integrates the functions of soothing the liver and harmonizing the stomach, as well as healing sores and promoting muscle growth. It aims to alleviate the symptoms of LSDCG through soothing the liver and regulating qi, strengthening the spleen and harmonizing the stomach, and promoting blood circulation and relieving pain (Xiaoyun et al., 2026; Zou et al., 2026). The main ingredients of WMS include *Bupleuri Radix*, *Aurantii Fructus*, *Angelicae Dahuricae Radix*, and *Bletillae Rhizoma*, etc. These drugs achieve therapeutic purposes by regulating the circulation of qi and blood in the liver meridian, intestinal meridian, and stomach meridian (Li et al., 2005; Wang et al., 2012). It is very important to further explore the mechanism of WMS in alleviating LSDCG.

This study aims to combine modern pharmacological viewpoints and TCM theories, and based on the gut microbiota-hepatic BA metabolism axis, explore the effect and mechanisms of WMS in treating LSDCG, providing a scientific basis for the development of novel therapeutic strategies for CG.

## 2 Materials and methods

### 2.1 Preparation of WMS

WMS is a clinical experience prescription developed by Chief Physician Junru Li, consisting of 20 g of Chai-hu (*Bupleuri Radix*), 16 g of Zhi-qiao (*Aurantii Fructus*), 10 g of Bai-zhi (*Angelicae Dahuricae Radix*), 16 g of Bai-ji (*Bletillae Rhizoma*), 5 g of San-qi (*Notoginseng Radix ET Rhizoma*), 6 g of Ru-xiang (*Olibanum*), 6 g of Mo-yao (*Myrrha*), 30 g of Wa-leng-zi (*Arcae Concha*), 30 g of Huang-qi (*Astragali Radix*), 10 g of Bing-lang (*Arecae Semen*), and 16 g of Bai-jiang-cao (*Herba Patriniae*), totaling 165 g per dose. All botanical drugs were authenticated by Prof. Junru Li according to the Chinese Pharmacopoeia (2025). The full species names, including authorities and family, are provided in Supplementary Table 1.

As described previously (Xiaoyun et al., 2026; Zou et al., 2026), all botanical drugs were sourced from a single GMP-certified supplier and provided by the TCM Pharmacy of Qinghai Provincial Hospital of Traditional Chinese Medicine. Voucher specimens were not deposited, as the materials were obtained through the hospital pharmacy′s routine clinical supply chain rather than field collection. Batch numbers, supplier quality-control reports, and purchase records are available from the hospital pharmacy upon request.

The preparation was performed at the Preparation Center of Qinghai Provincial Hospital of Traditional Chinese Medicine. Briefly, the botanical drugs were soaked in 8 volumes of distilled water (1 g crude drug: 8 mL water) and then decocted. After boiling, the decoction was simmered for 30 min and filtered. The residue was then decocted again with 6 volumes of distilled water (1 g crude drug: 6 mL water) for 20 min and filtered. The two filtrates were combined and concentrated. The extract yield was 13.8%, corresponding to a drug–extract ratio (DER) of approximately 7.25:1. The concentrated decoction was stored at 4 °C until use. The equivalent WMS dose for rats was calculated as 17.3 g/kg based on the adult clinical dosage using the body surface area conversion method (crude drug equivalent).

### 2.2 Animal experimental design

A total of 30 SPF SD male rats, weighing 230 ± 10 g, were provided by Chengdu Dashuo Experimental Animal Co., LTD (SCXK [chuan]2020–0030). Animal experiments were conducted under the conditions of a barrier system, temperature 20 ∼ 26 °C, relative humidity 30 ∼ 70%, 12 h day/12 h night light cycle. All experimental operations have been approved by the Animal Ethics Committee of Qinghai Provincial Hospital of Traditional Chinese Medicine (no. QZYEC20241223-021). After 7 days of adaptive feeding, the animals were randomly divided into 5 groups: control group, LSDCG model group, LSDCG + WMS treatment group, CG model group, and CG + WMS treatment group (n = 6). The sample size (n = 6 per group) was determined based on a combination of previous experimental experience (Xiaoyun et al., 2026; Zou et al., 2026), power calculations, and the 3Rs principle for animal ethics.

The rats were given 56% ethanol (8 g/kg) by intragastric administration every 3 days for 4 weeks to induce the CG model. After 3 weeks of gastritis modeling, 1/3 of the tails of the rats were clamped to induce aggression and fighting. The clamp position was changed once every 15 min to avoid injury caused by prolonged tail ischemia. The clamp was stimulated for 30 min a day for 7 days to form a model of LSDCG. After successful model establishment, the LSDCG + WMS and CG + WMS treatment groups were administered WMS by gavage at 34.6 g/kg/day (i.e., 2 × the clinical equivalent dose, crude drug equivalent) for 4 weeks, based on our previous experiments which indicated better efficacy at this dose (Xiaoyun et al., 2026; Zou et al., 2026). The control, LSDCG, and CG groups were given the same amount of normal saline for 4 weeks. The weight of the rats was measured weekly during the experiment. In addition, rats were evaluated based on the animal syndrome score sheet, which was divided into four parts: emotional condition, hair condition, fecal condition and food intake. Each part was scored from 0 to 3, and the total score was the sum of the four parts. The higher the score, the more obvious the LSD state. To minimize observer bias, all behavioral assessments were performed by two independent trained observers who were blinded to the group allocation, and the average of their scores was used for analysis.

At the end of the experimental period, all rats were fasted overnight (with free access to water) prior to euthanasia. Animals were deeply anesthetized by intraperitoneal injection of sodium pentobarbital at a dose of 50 mg/kg body weight. The depth of anesthesia was confirmed by the absence of pedal withdrawal reflex and corneal reflex, as well as by the loss of spontaneous breathing efforts. Following the loss of the corneal reflex and complete lack of response to toe pinch, the rats were euthanized by cervical dislocation. All efforts were made to minimize suffering, and the euthanasia procedures were performed by trained personnel in compliance with institutional animal welfare regulations.

### 2.3 Behavioral experiment

Open field experiments were conducted on rats at the end of drug administration. Before the test, the rats were moved to the behavioral room and the lights were turned off to adapt for 1 h. During the test, the rats were gently placed with their backs to the experimenter in the center of the experimental box with a length and width of 1 m each and freely explored for 5 min. The movement trajectories of each rat in the box were recorded by a camera, and the total distance and average speed of the rats were analyzed by animal behavior software.

### 2.4 Hematoxylin-eosin staining (HE)

The fixed gastric mucosa tissues of rats were dehydrated, embedded, sliced and dewaxed to water, stained with hematoxylin for 15 min, differentiated with hydrochloric acid alcohol for 5 s, returned to blue with warm water at 50 °C, then treated with 85% alcohol for 5 min and stained with eosin for 5 min. The gradient alcohol dehydration was performed, followed by xylene transparency, neutral gum sealing, and final microscopic observation.

### 2.5 Enzyme-linked immunosorbent assay (ELISA)

Blood samples were collected from each group, and serum was obtained by centrifugation at 3000 rpm for 15 min. ELISA kits (no. ZC-36391, ZC-36404, ZC-36386, ZC-37624, zcibio, China) were used to detect the levels of serum pro-inflammatory factor IL-6, TNF-α, IL-1β, and IL-17, respectively. Set up standard holes and sample holes, adding different concentrations of standard products and samples, respectively. Horseradish peroxidase-labeled antibody was detected and incubated at 37 °C for 60 min after washing the plate. Add substrate to each well and incubate at 37 °C for 15 min. The OD values of each hole were measured at 450 nm wavelength within 10 min after the termination solution was added.

### 2.6 AB-PAS staining

The tissue sections were dewaxed to water, then dripped with Alcian blue staining solution for 20 min, followed by oxidation with oxidizing agent for 5 min. Soak in Schiff dyeing solution for 10–20 min, and then dye the nuclei with hematoxylin solution for 1–2 min. Finally, the acid differentiation solution was differentiated for 2–5 s, Scott blue solution was returned to blue for 3 min.

### 2.7 Real-time quantitative polymerase chain reaction (RT-qPCR)

Total RNA was extracted from the gastric mucosa tissues of rats with Molpure Cell/Tissue Total RNA Kit (no. 19221ES50, YEASEN, China), and the genomic DNA was removed for reverse transcriptional reaction and converted into cDNA by PrimeScript RT reagent Kit (no. RR047A, TAKARA, China). Using cDNA as template, fluorescent quantitative PCR reaction was carried out with each gene primer and TB Green Premix Ex Taq II (Tli RNaseH Plus) Kit (no. RR820A, TAKARA, China), and the CT values of each detected sample were analyzed by QuantStudio Design & Analysis SE Software (Thermo, USA). The relative mRNA expressions of each gene were calculated by 2^-△△CT^. All primers were obtained from Sangon Biotech (China), and purified by ULTRAPAGE (Table 1).

**TABLE 1:** Sequence of primers used in the article.

Genes	Forward primer (5′–3′)	Reverse primer (5′–3′)
β-actin	GGG​AAA​TCG​TGC​GTG​ACA​TT	GCGGCAGTGGCCATCTC
ACADVL	CTT​GGA​GAA​GGT​GGA​GGA​GGA​C	AGC​GAG​CGT​ACT​GGG​TAT​TAG​AG
AOX-1	GAA​GTC​TTG​GTC​TCA​GTG​AAC​ATC​C	ATT​CCA​GAG​TTG​ACA​ATC​GCT​AGT​G
Claudin	CTT​CTG​GGT​TTC​ATC​CTG​GCT​TC	CCT​GAG​CAG​TCA​CGA​TGT​TGT​C
Cpt1a	AGA​CAG​ACA​CCA​TCC​AAC​ATA​TCA​AG	GCC​TCA​GCA​GCC​TCC​CAT​C
Cpt1b	CTC​CCA​TCA​TCG​GTC​ACC​TCT​G	AGGCGGCGGCAACTTGG
E-cadherin	AGG​AGG​TCT​TTG​AGG​GAT​CTG​TTG	ATC​TTG​GCT​GAG​GAT​GGT​GTA​GG
H^+^-K^+^ATPase	TCA​CGG​CGG​ACA​TGC​TAC​AG	GCT​GGG​CAG​GAA​CTT​GAC​AAT​C
Intelectin-1	TGC​CAC​AAG​TGA​TGA​CTA​CAA​GAA​C	AGC​CAG​TGA​AGG​TGC​GGT​AC
Muc5ac	TCA​CCA​TCG​CCA​CTC​TGT​CAG	AGC​ACA​CCA​CCT​CCA​AGT​CC
Muc6	TAC​CAG​CCA​AGT​GAC​GCA​GAC	GAG​ACC​GAG​GAG​CCC​AGA​TTG
Occludin	TCG​TGA​TGT​GCA​TCG​CTG​TAT​TC	CCT​CCG​AAG​CCA​CCT​CCA​TAG
Pgc	ACT​TAC​TAC​ACC​GAA​GGG​CAG​AC	ACC​AGG​CTC​ATT​CTC​ACT​CAG​G
Sst	GCT​GAG​CAG​GAC​GAG​ATG​AGG	AAC​AGG​ATG​TGA​ATG​TCT​TCC​AGA​AG
ZO-1	GCG​GAT​GGT​GCT​ACA​AGT​GAT​G	GCC​TTC​TGT​ATC​TGT​GTC​TTC​ATA​GTC

### 2.8 16S rRNA gene sequencing

The colonic contents of rats in each group were collected, total DNA was isolated, the DNA concentration was measured using Nanodrop, and the quality of the DNA extraction was assessed through agarose gel electrophoresis. The target sequences reflecting the composition and diversity of bacterial flora were taken as targets, primers were designed based on the conserved regions within these sequences, and the specific Barcode sequences were added for PCR amplification. The amplified products were purified by magnetic beads and quantified by PCR. The sequencing libraries were prepared using the Illumina TruSeq Nano DNA LT Library Prep Kit. Before on-machine sequencing, the library was analyzed on Agilent Bioanalyzer with the Agilent High Sensitivity DNA Kit. Then high-throughput sequencing was performed on the computer to analyze the differences in bacterial composition.

### 2.9 BAs metabolomics analysis

Colon contents and serum samples were collected for targeted metabolomics analysis. The BA standard was accurately weighed, and the final concentration of the mixed standard mother liquor was prepared with methanol to 1000 μg/mL, and then ten standard curve points were diluted with 30% methanol. The samples were subjected to protein precipitation, diluted with pre-cooled methanol and passed through a 0.22 μm filter membrane for detection. The chromatographic analysis was completed using the ACQUITY UPLC BEH C18 column (2.1 × 100 mm, 1.7 μm, Waters, USA), and mass spectrometry detection was carried out using the electrospray ionization (ESI) source and the negative ion ionization mode. The mixed standard sample, the control sample, and the sample to be tested were measured successively. The standard curve was drawn according to the peak area of the standard sample, followed by linear regression analysis for quantification and data conversion.

### 2.10 Immunohistochemical staining

Paraffin sections were dewaxed to water, repaired in microwave oven for 20 min, and incubated in 3% hydrogen peroxide at room temperature for 25 min to block endogenous peroxidase. Bovine serum (no. GC305010, Servicebio, China) was incubated for 20 min, incubated with PPARα (no. 16643-1-AP, Proteintech, China) and RXR (no. bs-1379R, Bioss, China) antibodies at 4 °C overnight, and 37 °C for secondary antibody (no. GB22303, Servicebio, China) for 30 min. After DAB (no. ZL1-9018, zsbio, China) was used for color development at room temperature, restain the nucleus with hematoxylin for 3 min, and then washed. Microscopic observation was carried out after conventional dehydration sealing.

### 2.11 Western blot analysis

The gastric mucosa tissues of rats were lysed with RIPA lysate (no. BL509A, Biosharp, China). After centrifugation, the proteins were collected and quantified using BCA kit (no. P0009, Beyotime, China). The protein concentration of each experimental group was adjusted, and the protein denaturation was carried out at high temperature. The denatured proteins were separated by SDS-PAGE and then transferred onto the PVDF membrane (no. ISEQ00010, Sigma-Aldrich, USA). It was then sealed with 5% skim milk (no. GC310001, Servicebio, China) for 2 h. PPARα (1:2000; no. AF5301, affbiotech, China), RXR (1:2000; no. A19105, abclonal, China), and β-actin (1:50,000; no. AC026, abclonal, China) antibodies were incubated overnight at 4 °C. Secondary antibody (1:5000; no. S0001, affbiotech, China) was incubated at room temperature for 2 h. Finally, the strips were exposed with ECL luminescent solution (no. 17046, zen-bio, China) and Tanon fluorescence image analysis system, and the exposure results were scanned by Gel-Pro analyzer software.

### 2.12 Immunofluorescent staining

Consistent with the pretreatment in the immunohistochemical experiment, paraffin sections of rat gastric mucosa were dewaxed to water, repair by microwave, blocked endogenous peroxidase, and then incubated with bovine serum, followed by immunofluorescence staining. Single stain: Primary antibody Lgr5 (no. bs-20747R, Bioss, China) was reacted at 4 °C overnight, and FITC conjugated antibody (no. GB22303, Servicebio, China) was reacted at 37 °C for 30 min. Double staining: The first primary antibody Lgr5 was incubated at 4 °C overnight, the corresponding HRP-labeled antibody (no. GB23303, Servicebio, China) was added and incubated for 50 min. TSA (FITC-Tyramide; no. G1222, Servicebio, China) was added and reacted for 10 min. The slices were immersed in the repair solution and microwave oven for antigen repair. The second antibody PPARα (no. ZRB237, Sigma, China), CPT1A (no. 15184-1-AP, Proteintech, China), or VLCAD (no. 14527-1-AP, Proteintech, China) was added and reacted at 4 °C overnight. The second antibody of the corresponding second antibody (no. GB21303, Servicebio, China) was reacted at 37 °C for 30 min. DAPI was used to reacted at room temperature for 10 min. Finally, the anti-fluorescence quenching sealing tablets were sealed and photographed under fluorescence microscope.

### 2.13 Statistical analysis

All experimental data were analyzed using SPSS software and are presented as mean ± standard deviation (SD). The normality of data distribution was assessed using the Shapiro–Wilk test, and homogeneity of variances was assessed using Levene′s test. All datasets met the assumptions of normality and equal variances (*P* > 0.05 for both tests). Therefore, parametric tests were applied. One-way analysis of variance (ANOVA) was used for comparisons among multiple groups. When a significant difference was detected by ANOVA, Tukey’s honestly significant difference (HSD) test was used for post-hoc pairwise multiple comparisons. Independent-samples t-test was used for two-group comparisons where indicated. Spearman correlation analysis was performed to assess the associations between the differential microbial genera (genus level) and the differential BAs in colonic contents and serum using R software. *P* < 0.05 was considered statistically significant.

## 3 Results

### 3.1 WMS improved the LSD syndrome and gastric mucosal inflammation in model rats

As shown in Figure 1A, the body weight of rats in both the LSDCG and CG model groups was lower than that in the control group, while the body weight of model rats after WMS treatment increased somewhat, but the difference was not statistically significant (*P* > 0.05). The syndrome score results showed that compared with the control group, the rats in the model group exhibited reduced diet, irritability and agitation, drowsiness or depression, dull skin and fur color, loose and soft stools, and the syndrome score significantly increased (*P* < 0.01, Figure 1B). Among them, the score of the LSDCG model group was significantly higher than that of the CG group (*P* < 0.001, Figure 1B). After WMS treatment, the dietary activity of rats increased, fur was lustrous, stools were normal, and the symptom score was significantly reduced (*P* < 0.05, Figure 1B). During the administration period, no diarrhea, piloerection, arched back, or abnormal behavior was observed in the WMS treatment group. Similarly, the behavioral changes of rats were analyzed through the open field experiment, found that the moving distance and average speed of rats in the LSDCG and CG model groups were significantly reduced (*P* < 0.05, Figures 1C,D). After WMS treatment, the moving distance and average speed of rats in the LSDCG and CG model groups were pointedly increased (*P* < 0.001, Figures 1C,D). HE staining displayed that in the LSDCG and CG model groups, a large number of cells atrophied, shrank, necrotic, and disappeared in the mucosal layer, leaving only a large number of residual cavities of the gastric glands. There was a slight inflammatory cell infiltration, mainly lymphocytes. Fibrous tissue hyperplasia was observed in some tissues, and a small amount of bleeding was also seen in the gastric mucosal layer of some tissues. The pathological damage degree of LSDCG rats was more severe than that of the CG group, and WMS alleviated the pathological damage of the gastric mucosal tissue in the LSDCG and CG model group rats (Figure 1E). In addition, the expressions of inflammatory factor indicators IL-1β, IL-6, IL-17, and TNF-α of rats in the LSDCG and CG model groups were significantly increased (*P* < 0.001, Figure 1F). The expressions of IL-1β and TNF-α were higher in the LSDCG group than in the CG group (*P* < 0.05, Figure 1F). WMS significantly reduced the levels of inflammatory factors in the serum of rats in the LSDCG and CG model groups (*P* < 0.01, Figure 1F).

**FIGURE 1:**
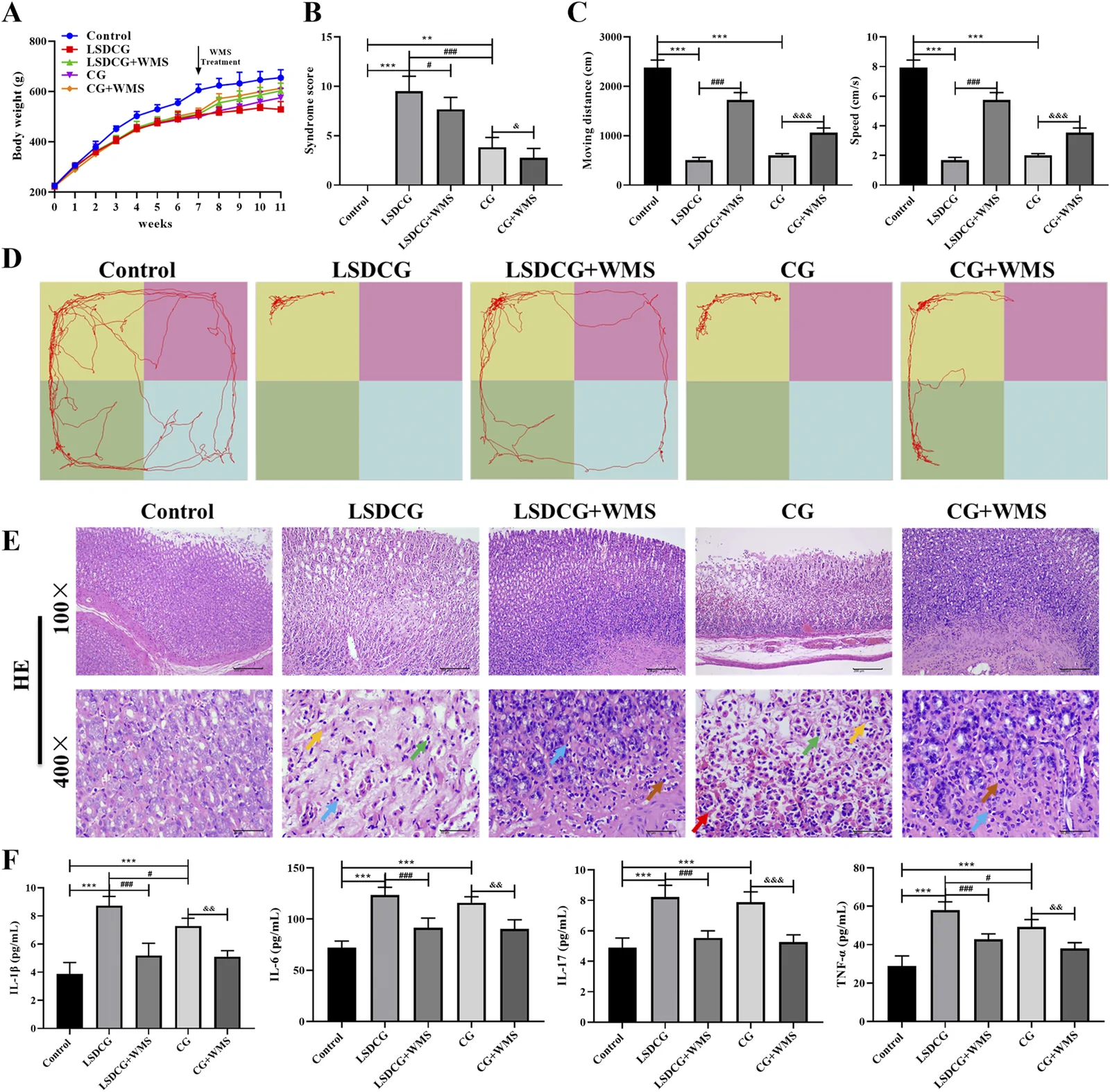
WMS improved the LSD syndrome and gastric mucosal inflammation in the model rats. **(A)** The body weight of rats was measured every week. **(B)** Statistical result chart of syndrome scoring in rats. **(C)** The total moving distance and average moving speed of the rats in the open field experiment. **(D)** Trajectory diagram of the rat in the open field experiment. **(E)** H&E staining was used to observe the pathological changes of gastric mucosal tissue in rats. The green arrows point to cell atrophy, the yellow arrows point to fibrous tissue, the blue arrows point to lymphocytes, the red arrows point to red blood cells, and the brown arrows point to neutrophils. Magnification: ×100 and 400×. **(F)** ELISA was used to detect the contents of inflammatory factors IL-1β, IL-6, IL-17, and TNF-α in serum. ^**^ *P* < 0.01, ^***^ *P* < 0.001, compared with the control group; ^#^ *P* < 0.05, ^###^ *P* < 0.001, compared with the LSDCG model group; ^&^ *P* < 0.05, ^&&^ *P* < 0.01, ^&&&^ *P* < 0.001, compared with the CG model group.

### 3.2 WMS improved the gastric mucosal barrier disruption in model rats

The AB-PAS staining results showed that the positive expression area of AB-PAS staining in rats of the LSDCG and CG model groups was significantly reduced, and it was significantly increased after WMS treatment (*P* < 0.01, Figures 2A,B). Similarly, compared with the control group, the mRNA levels of occludin, claudin, zo-1, E-cadherin in the gastric mucosal, and the antibacterial factor intelectin-1 in rats of the LSDCG and CG model groups were significantly decreased (*P* < 0.01, Figure 2C). After WMS treatment, the expression of these genes in rats of the LSDCG and CG model groups was significantly increased (*P* < 0.05, Figure 2C). It was suggested that WMS improved the gastric mucosal barrier disruption in model rats.

**FIGURE 2:**
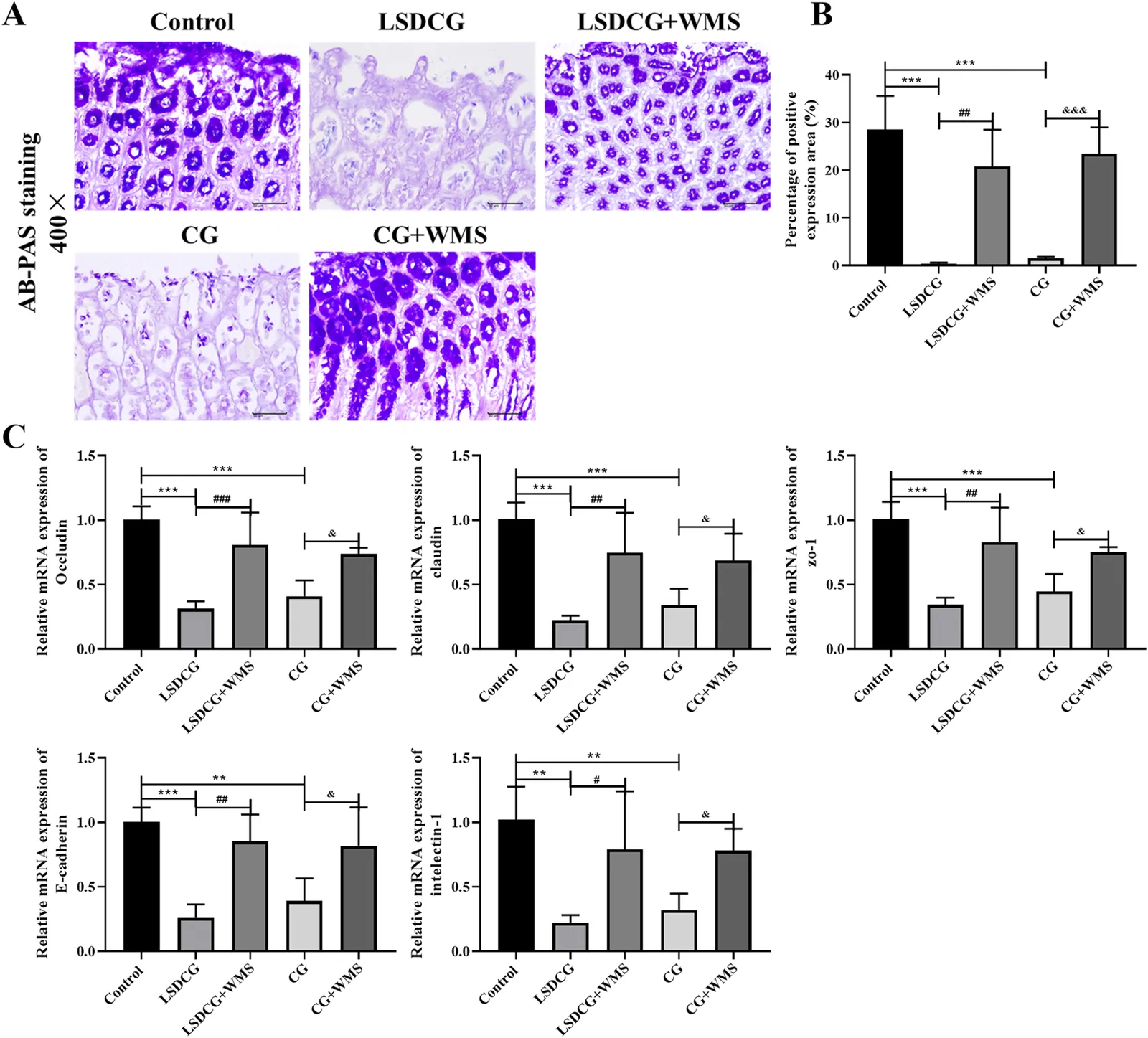
WMS improved the disruption of the gastric mucosal barrier in model rats. **(A)** AB-PAS staining was used to observe the distribution of mucin in the gastric mucosa of rats. **(B)** Statistical results of the positive expression area of AB-PAS staining. **(C)** The mRNA levels of gastric mucosal tight junction proteins occludin, claudin, zo-1, E-cadherin, and the antibacterial factor intelectin-1 were detected by RT-qPCR. ^**^ *P* < 0.01, ^***^ *P* < 0.001, compared with the control group; ^#^ *P* < 0.05, ^##^ *P* < 0.01, ^###^ *P* < 0.001, compared with the LSDCG model group; ^&^ *P* < 0.05, ^&&&^ *P* < 0.001, compared with the CG model group.

### 3.3 WMS regulated the intestinal microbiota of model rats

The intestinal microbiota of rats in each group was sequenced, and the sample abundance values within each group of each species were normalized. The species richness of each group at the genus level is shown in Figure 3A. Community analysis was conducted using a Venn diagram. Each ellipse represents a group, and the overlapping areas among the ellipses indicate the common ASV/OTU among the groups. As shown in Figure 3B, there are a total of 187 (2.17%) bacterial communities at the intersection of the five groups. To further compare the differences in species composition among the samples, UPGMA cluster analysis was conducted on the species composition data based on Euclidean distance. The heatmap of the composition of the bicluster genus-level species composition is shown in Figure 3C.

**FIGURE 3:**
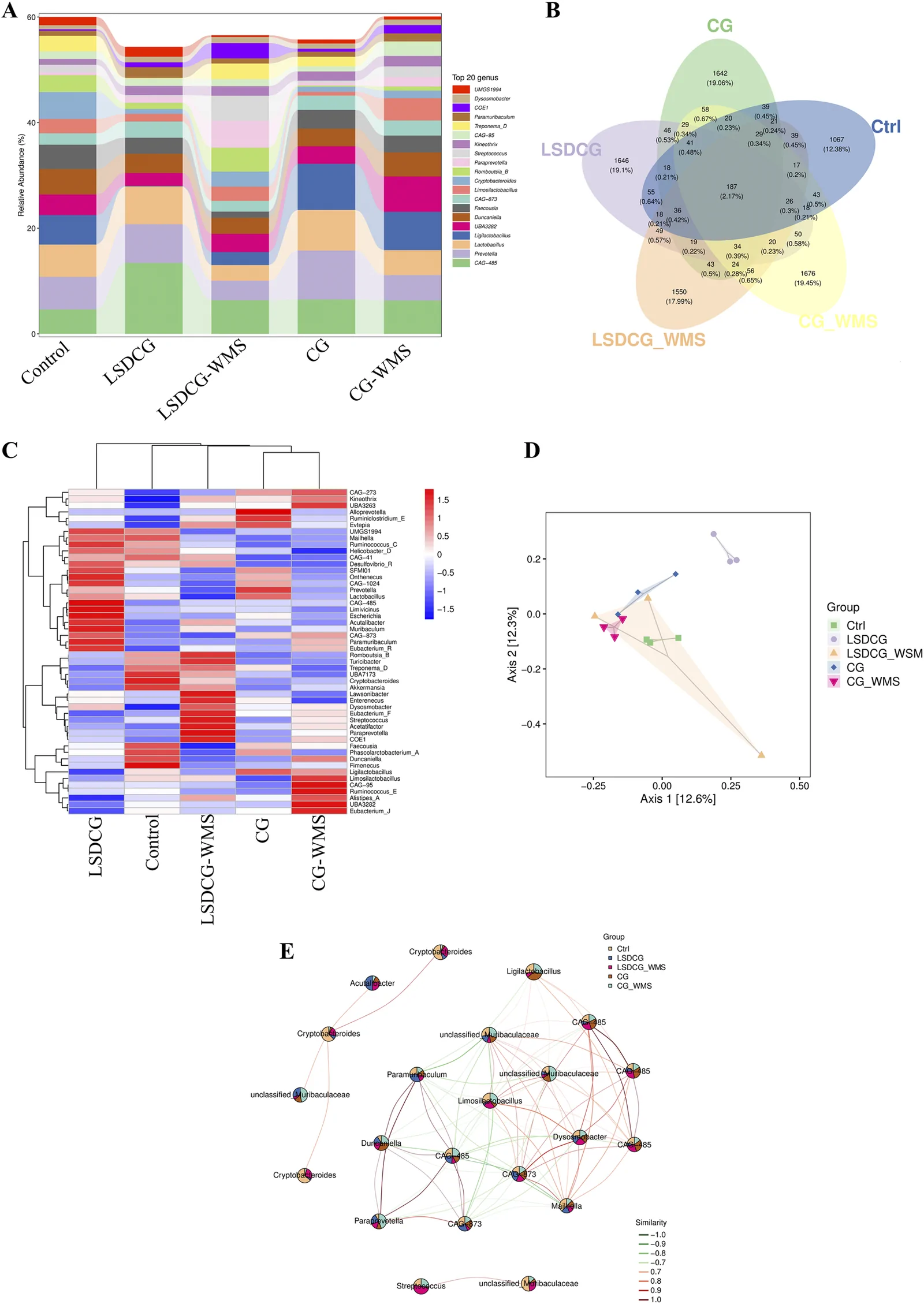
The 16S rRNA gene sequencing of the microbiota in colonic contents. **(A)** Species composition chart at the genus level. **(B)** Venn diagram. **(C)** Heatmap of species composition at the genus level of biclustering. **(D)** Principal coordinate analysis (PCoA) plot based on Bray-Curtis distance. **(E)** Dominant subnetwork diagram with grouped abundance pie chart.

Based on this clustering, we next assessed the diversity and structural differences of the microbial communities among groups. The α diversity analysis showed no significant differences in Chao1, Shannon, and Simpson indices among the five groups (*P* > 0.05). A marginal significance (*P* = 0.05) was observed for Chao1 between the control and LSDCG groups. Although no significant differences were observed in α diversity indices, β diversity analysis revealed a clear separation in microbial community structure among groups. As shown in Figure 3D, principal coordinate analysis (PCoA) based on Bray-Curtis distance showed that the first two principal coordinates explained 12.6% and 12.3% of the total variation, respectively. PERMANOVA confirmed a significant difference among the five groups (R^2^ = 0.352, *P* = 0.001), indicating that between-group differences exceeded within-group variation (Table 2). Based on the abundance of nodes (ASV/OTU), the top 50 nodes with the highest average abundance are extracted to construct the dominant object subnetwork. The dominant object subnetwork diagram with a grouped abundance pie chart is shown in Figure 3E. It mainly involves *Cryptobacteroides*, *Acutalibacter*, unclassified_Muribaculaceae, *Ligilactobacillus*, *Paramuribaculum*, *Duncaniella*, *Paraprevotella*, *Limosilactobacillus*, CAG-485, *Dysosmobacter*, CAG-873, and *Mailhella*, etc.

**TABLE 2:** PERMANOVA/adonis results for microbial community composition based on Bray-Curtis distance.

Source	df	SumofSqs	Pseudo-F	R^2^	p-value (perm = 999)
Group	4	1.409	1.358	**0.352**	**0.001**
Residual	10	2.594	—	0.648	—
Total	14	4.003	—	1.000	-

Bold values indicate statistically significant PERMANOVA results (R^2^ = 0.352, P = 0.001).

### 3.4 WMS regulated the BA metabolism of model rats

Further analysis of BA metabolism in the colon and serum was conducted using metabolomics. The metabolic changes of BAs in colonic contents are shown in Figure 4, and those in serum are shown in Figure 5. To screen the differential substances in each comparison dimension, the multivariate statistical method principal components analysis (PCA) was used to analyze different comparison dimensions. As shown in Figures 4A, 5A, there are certain differences in the BA metabolic levels both in the colonic contents and serum of each group. The Pearson correlation coefficient was used to measure the linear correlation between the two variables. As shown in Figures 4B, 5B, the Pearson correlation coefficients of each sample are close to 1, indicating that the expression trends of metabolites in the two samples are similar. Further overall heatmap analysis was conducted on all samples and the detected metabolites (Figures 4C, 5C). Figures 4D, 5D present the BA differences of each comparison group in the form of a Venn diagram. Among the colonic contents, the common difference in BAs between control vs. LSDCG, LSDCG vs. LSDCG-WMS, and CG vs. LSDCG is taurohyodeoxycholic acid sodium salt (THDCA) + tauroursodeoxycholic acid sodium salt (TUDCA). Chenodeoxycholic acid (CDCA) and Tauro-α-muricholic acid sodium salt (T-α-MCA) are common differences between CG vs. LSDCG, and LSDCG vs. LSDCG-WMS. The difference BAs in the control vs. CG is hyodeoxycholic acid (HDCA) (Figure 4D). And in the serum, the difference between LSDCG vs. LSDCG-WMS is deoxycholic acid (DCA), the control vs. LSDCG are CDCA and chenodeoxycholic acid-glucuronic acid conjugate (CDCA-G), control vs. CG are CDCA, CDCA-G, allolithocholic acid (alloLCA), 23-Nordeoxycholic acid (NorDCA), and ursocholic acid (UCA). The difference BAs between CG vs. CG-WMS is NorDCA, and between CG-WMS vs. LSDCG-WMS are DCA, NorDCA, lithocholic acid (LCA), and taurodeoxycholic acid sodium salt (TDCA). Moreover, the KEGG enrichment results presented that the differentially expressed BAs in the colonic contents and serum are both enriched in “Bile secretion” and “Primary bile acid biosynthesis” (Figures 4E, 5E).

**FIGURE 4:**
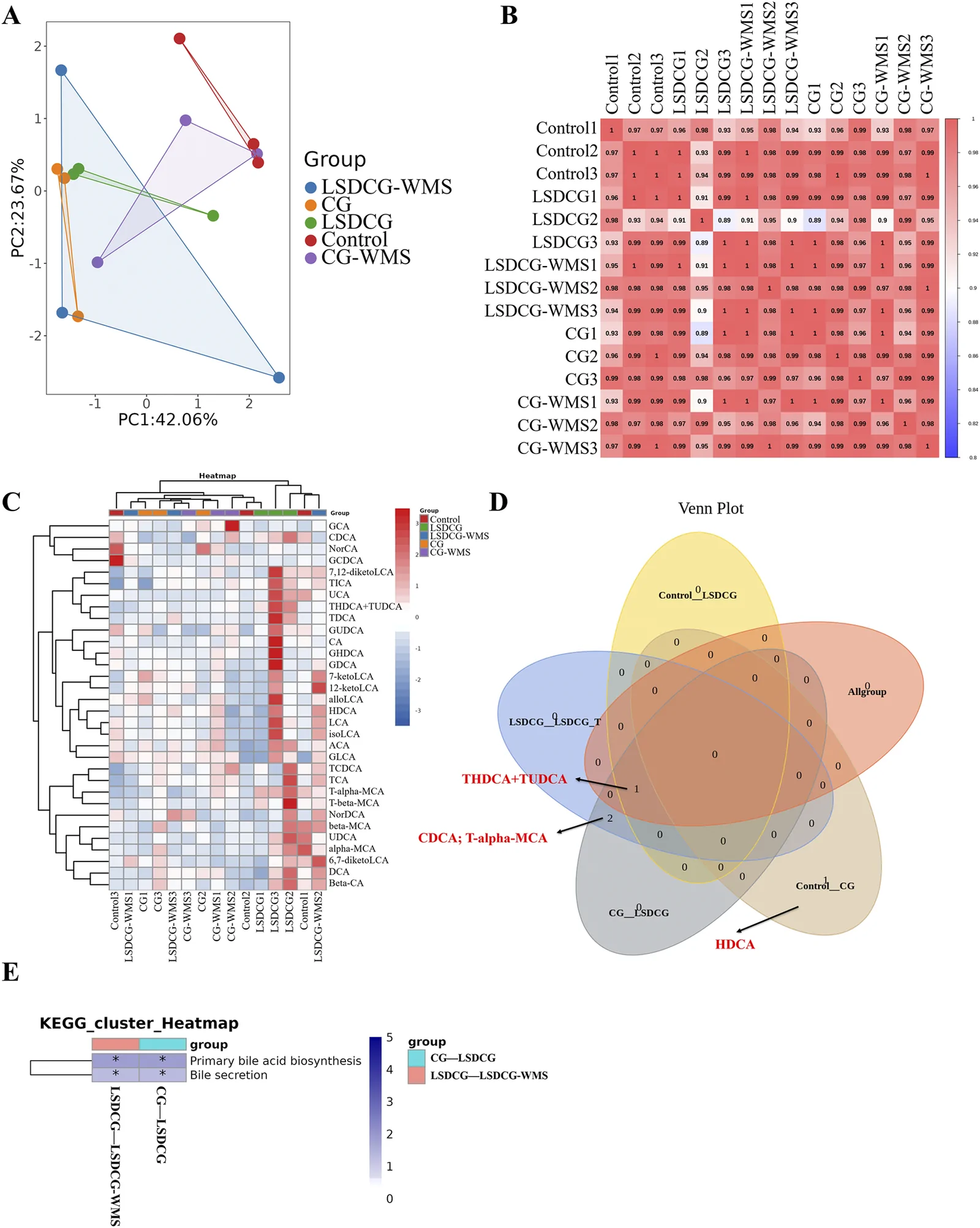
Metabolic changes of bile acids (BAs) in colonic contents. **(A)** Principal components analysis (PCA). **(B)** Sample correlation analysis. **(C)** Metabolite heatmap. **(D)** Venn diagram. **(E)** KEGG enrichment pathway.

**FIGURE 5:**
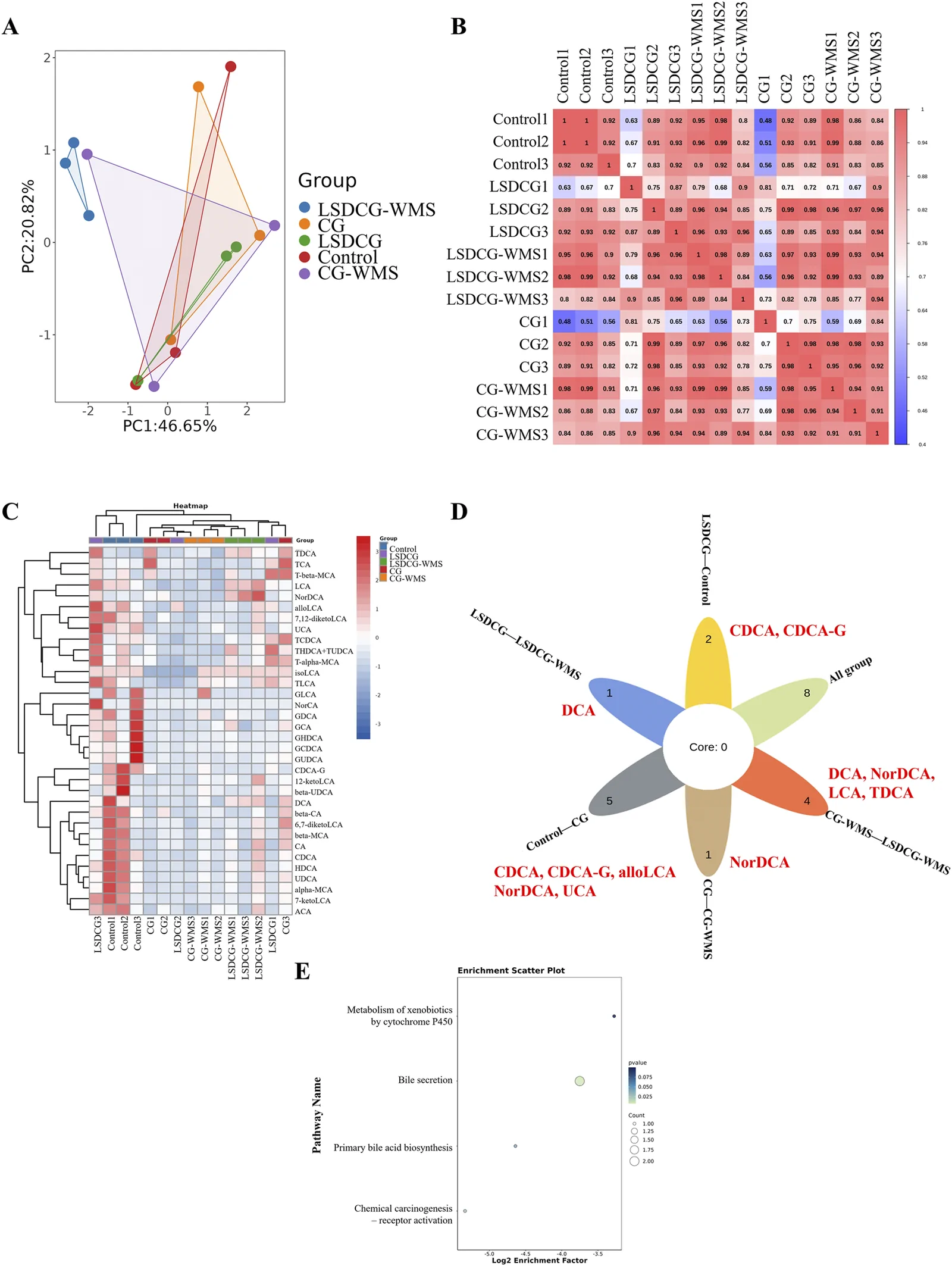
Metabolic changes of BAs in serum. **(A)** Principal components analysis (PCA) analysis. **(B)** Sample correlation analysis. **(C)** Metabolite heatmap. **(D)** Venn Diagram. **(E)** KEGG enrichment pathway.

### 3.5 Correlation between intestinal microbiota and BA metabolism

To explore the potential association between intestinal microbiota and BA metabolism, Spearman correlation analysis was performed between the differential microbial genera and the BAs (Supplementary Figure 1). In colonic contents, several genera showed nominal associations with BAs, including negative correlations between *Duncaniella* and 12-ketoLCA, β-CA, 7,12-diketoLCA, DCA, 7-ketoLCA, alpha-MCA, and beta-MCA; negative correlations between unclassified_Muribaculaceae and 12-ketoLCA, 7-ketoLCA, alloLCA; negative correlations between COE1 and UDCA; negative correlations between Kineothrix and UDCA, alpha-MCA, and beta-MCA; negative correlations between unclassified_Lachnospiraceae and UDCA, alpha-MCA, and beta-MCA; positive correlations between Faecousia and alpha-MCA, and beta-MCA; positive correlations between Romboutsia_B and UDCA, alpha-MCA, and beta-MCA, etc. (*P* < 0.05, Supplementary Figure 1A). In serum, several associations remained significant, including a strong negative correlation between COE1 and ACA (*P* < 0.001) and between unclassified_Lachnospiraceae and ACA (*P* < 0.001, Supplementary Figure 1B), etc.

### 3.6 WMS regulated the expression of BA receptors in the gastric tissues of model rats

The metabolomics results showed that WMS could significantly regulate the BAs levels of model rats, so the BA receptors were analyzed. The immunohistochemical results disclosed that, the expression levels of BA receptors PPARα and RXR in the gastric tissues of rats in the LSDCG and CG model groups were significantly decreased, and the expression level of RXR in the LSDCG group was lower than that in the CG group (*P* < 0.001, Figures 6A–C). After WMS treatment, the decreased levels in the LSDCG group was significantly increased (*P* < 0.001, Figures 6A–C). Similarly, the WB results also showed that the expressions of PPARα and RXR in the gastric tissues of rats in the LSDCG and CG model groups were significantly decreased, their expressions in the LSDCG group were both lower than those in the CG group, and WMS significantly increased their expression (*P* < 0.05, Figures 6D,E).

**FIGURE 6:**
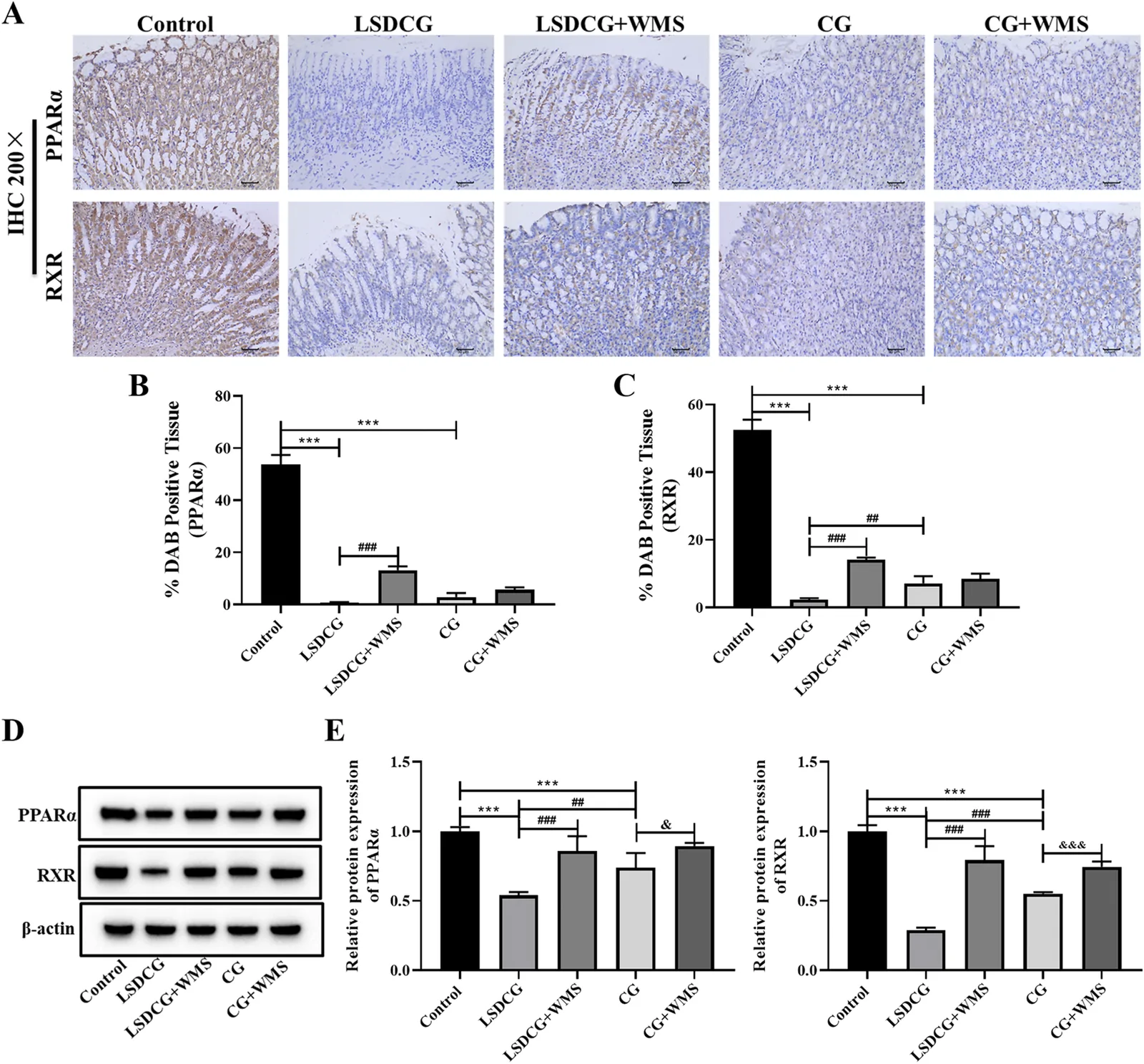
WMS regulated the expression of BA receptors in the gastric tissue of model rats. **(A)** Immunohistochemical staining was used to detect the expression of BA receptors PPARα and RXR in the gastric tissue. Magnification ×200. **(B)** Statistical results of positive expression of PPARα in the tissues. **(C)** Statistical results of positive expression of RXR in the tissue. **(D)** WB was used to detect the protein expression of PPARα and RXR in gastric tissues. **(E)** Statistical results of the relative protein expression levels of PPARα and RXR. ^***^ *P* < 0.001, compared with the control group; ^##^ *P* < 0.01, ^###^ *P* < 0.001, compared with the LSDCG model group; ^&^ *P* < 0.05, ^&&&^ *P* < 0.001, compared with the CG model group.

### 3.7 WMS reversed the loss and functional impairment of Lgr5-positive gastric stem cells in model rats

The staining results of Lgr5 positive (Lgr5+) cells displayed that the expression level of Lgr5 in the gastric tissues of rats in the LSDCG and CG model groups was significantly lower than that in the control group. Among them, the LSDCG group was lower than the CG group, and WMS significantly increased the number of Lgr5+ cells (*P* < 0.001, Figures 7A,B). Furthermore, the RT-qPCR detection results showed that compared with the control group, the mRNA expressions of surface mucous cell marker Muc5ac, parietal cell marker H + -K + ATPase, chief cell marker Pgc, mucous neck cell marker Muc6 and endocrine cell marker Sst in the gastric tissues of rats in the LSDCG and CG model groups were significantly decreased, while WMS treatment significantly promoted their expression (*P* < 0.05, Figure 7C).

**FIGURE 7:**
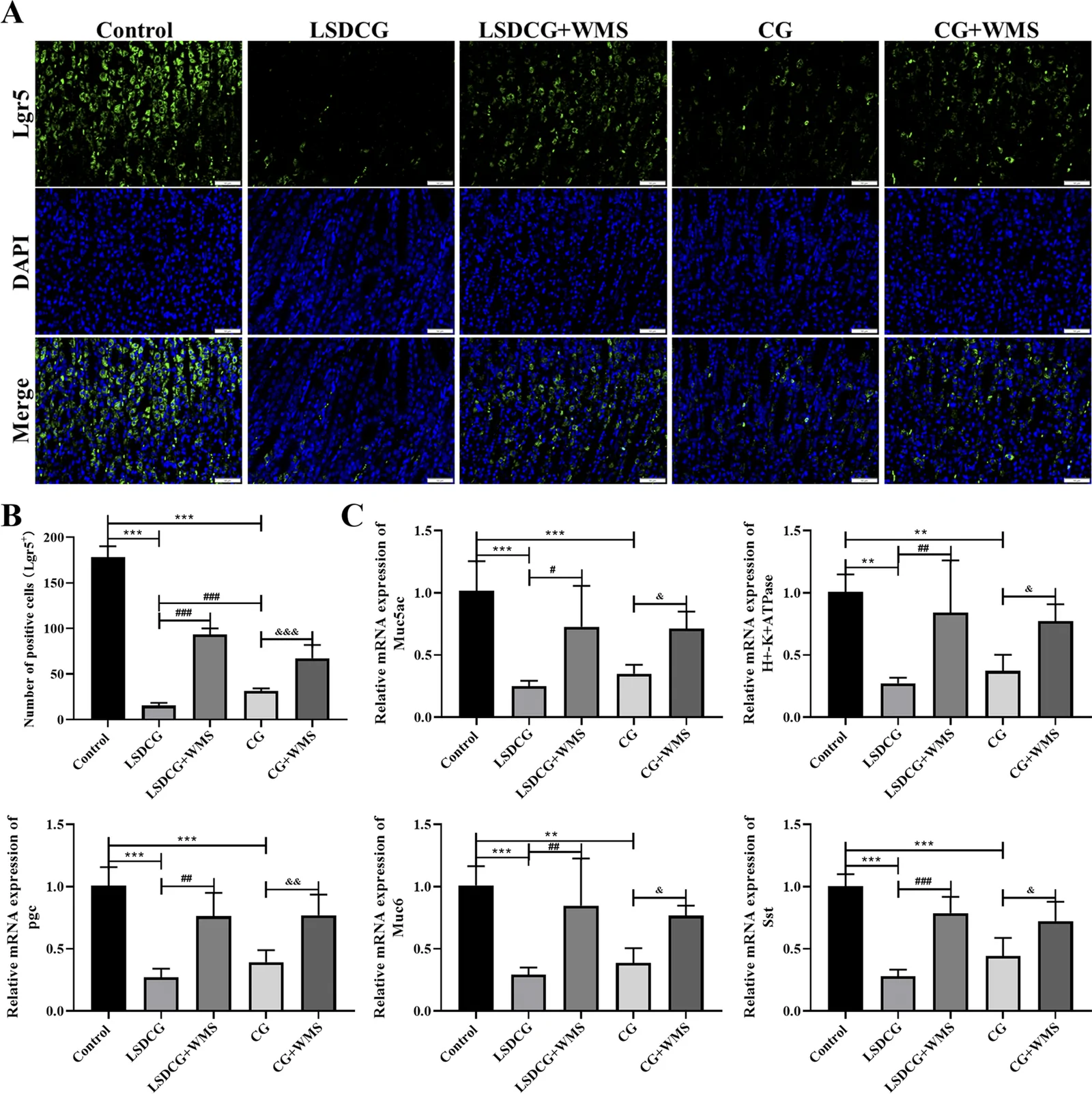
WMS reversed the loss and functional impairment of Lgr5+ gastric stem cells in model rats. **(A)** Immunofluorescence staining of Lgr5 was used to observe the distribution of Lgr5^+^ gastric stem cells. **(B)** Statistical results of the number of Lgr5^+^ cells. **(C)** RT-qPCR was used to detect the expression of differentiation markers of gastric mucosal cells: surface mucous cells (Muc5ac), parietal cells (H + -K + ATPase), chief cell (Pgc), mucous neck cell (Muc6), and endocrine cells (Sst). ^**^ *P* < 0.01, ^***^ *P* < 0.001, compared with the control group; ^#^ *P* < 0.05, ^##^ *P* < 0.01, ^###^ *P* < 0.001, compared with the LSDCG model group; ^&^ *P* < 0.05, ^&&^ *P* < 0.01, ^&&&^ *P* < 0.001, compared with the CG model group.

### 3.8 WMS regulated fatty acid oxidation in Lgr5+ gastric stem cells of model rats

Lgr5+ stem cells are a vital cell population for keeping the self-renewal of gastric epithelium, and their dysfunction may lead to damage of the gastric mucosal barrier (Barker et al., 2010). Existing studies have shown that BAs are related to the activation of Lgr5+ stem cells and epithelial regeneration (Sorrentino et al., 2020). We observed the expression of Lgr5+ stem cells through immunofluorescence staining. The results found that the number of cells co-expressing PPARα within Lgr5+ gastric stem cells (Lgr5+PPARα+) was significantly reduced in the LSDCG and CG model groups compared to controls. This reduction was more pronounced in the LSDCG group than in the CG group, whereas WMS intervention significantly increased its expression (*P* < 0.001, Figures 8A,B). Similarly, the numbers of cells positive for carnitine palmitoyl-transferase1A (CPT1A) and very long-chain acyl-CoA dehydrogenase (VLCAD) within Lgr5+ gastric stem cells (Lgr5+CPT1A+ and Lgr5+VLCAD+) were also significantly decreased in both the LSDCG and CG groups (*P* < 0.001). Notably, the number of Lgr5+CPT1A + cells was significantly lower in the LSDCG group than in the CG group, and WMS treatment significantly increased the number of Lgr5+CPT1A+ and Lgr5+VLCAD + cells in the LSDCG group, whereas in the CG group, it only promoted the number of Lgr5+CPT1A + cells (*P* < 0.001, Figures 8C–F). In addition, the gene expression levels of AOX-1, Cpt1a, Cpt1b, and ACADVL in the gastric tissues of rats in the LSDCG and CG model groups were significantly decreased, and their expressions were significantly promoted after WMS treatment (*P* < 0.001, Figure 8G).

**FIGURE 8:**
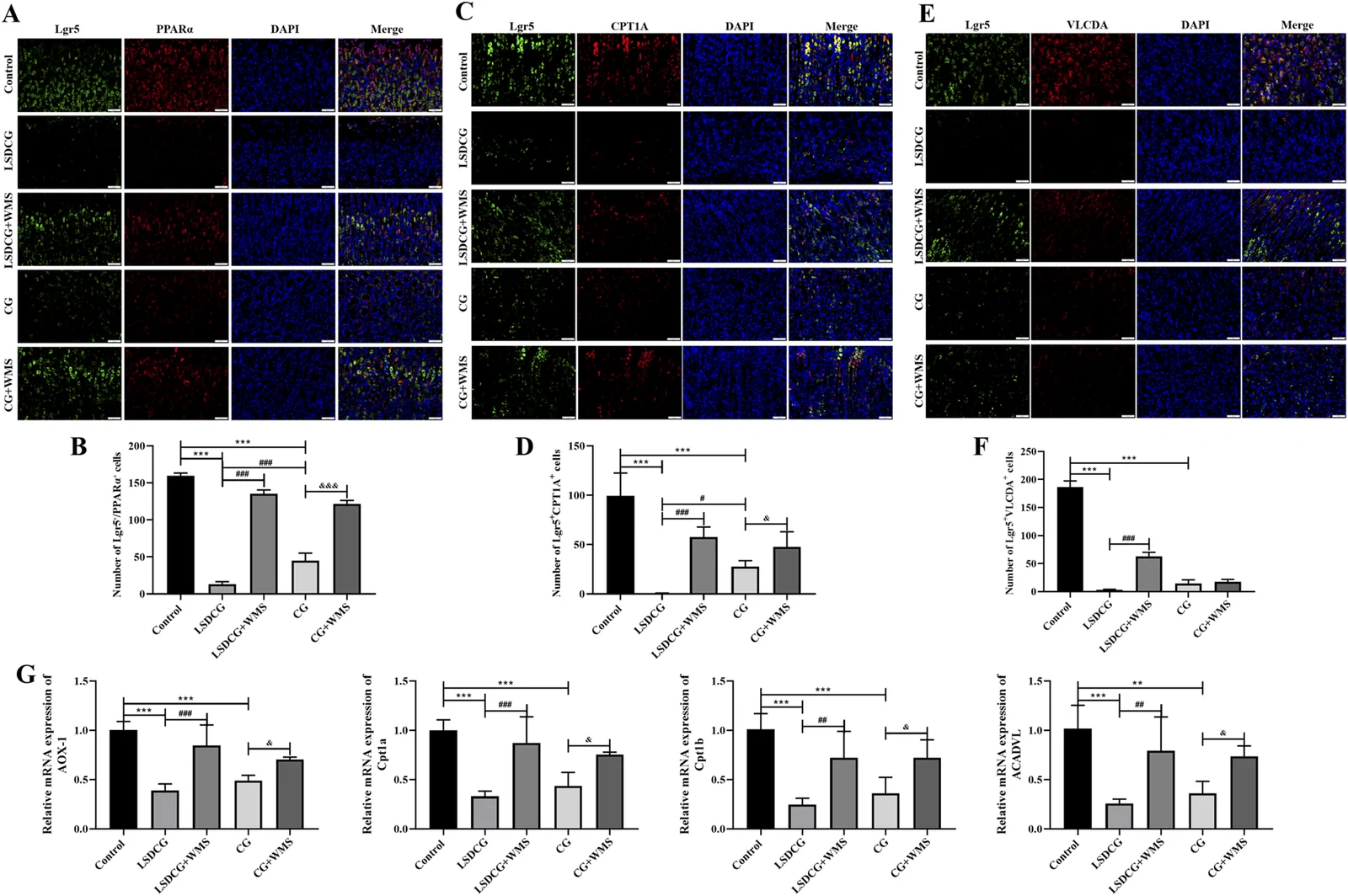
WMS regulated fatty acid oxidation in Lgr5+ gastric stem cells of model rats. **(A)** Double immunofluorescence staining of Lgr5 and PPARα. **(B)** Number of Lgr5^+^PPARα^+^ double-positive cells. **(C)** Double immunofluorescence staining of Lgr5 and CPT1A. **(D)** Number of Lgr5^+^CPT1A^+^ double-positive cells. **(E)** Double immunofluorescence staining of Lgr5 and VLCAD. **(F)** Number of Lgr5^+^VLCAD^+^ double-positive cells. **(G)** RT-qPCR was used to detect the gene expressions of AOX-1, Cpt1a, Cpt1b, and ACADVL in gastric tissues. ^**^ *P* < 0.01, ^***^ *P* < 0.001, compared with the control group; ^#^ *P* < 0.05, ^##^ *P* < 0.01, ^###^ *P* < 0.001, compared with the LSDCG model group; ^&^ *P* < 0.05, ^&&&^ *P* < 0.001, compared with the CG model group.

## 4 Discussion

CG is a type of chronic inflammatory lesion of the gastric mucosa caused by various etiologies. Its incidence rate ranks first among all kinds of gastric diseases, and it is characterized by repeated attacks and prolonged illness (Osadchuk et al., 2016). Among the various causes of CG, the dysfunction of the liver, spleen, and stomach plays an important part. The type of LSD is one of the main syndrome types of CG in TCM (Zhang et al., 2019; Bai et al., 2024). WMS is a clinical empirical formula developed by Chief Physician Junru Li at our institution. Preliminary research has confirmed that it can effectively inhibit systemic inflammation and repair gastric mucosal damage for patients with chronic erosive gastritis, gastric ulcers, and other gastrointestinal diseases (Guan et al., 2024). Our research found that both the CG and LSDCG model rats exhibited characteristics such as weight loss and increased TCM syndrome scores. The TCM syndrome score of the LSDCG group was significantly higher than that of the CG group, showing more significant characteristics such as reduced diet, irritability and agitation, drowsiness or depression, slowed weight gain, dull skin and fur color, and loose and soft stools. WMS significantly improved the TCM syndrome scores of CG and LSDCG model rats, manifested as increased diet, increased body weight, lustrous fur color, and normal stool in rats. Similarly, WMS increased the autonomous motor ability, inhibited inflammatory responses, and improved gastric mucosal injury and mucosal barrier disruption of model rats.

At present, there have been many scientific understandings of the pathogenic mechanism of CG. Anxiety and depression are common mental illness complications among patients with chronic digestive tract diseases (Andriolo et al., 2024). In a study involving 1641 patients with gastrointestinal diseases, it was found that 1379 cases presented with a state anxiety, 1098 cases presented with trait anxiety, and 442 cases presented with a current depression (Addolorato et al., 2008). Whether in patients with functional gastrointestinal diseases or those with organic injuries, the concomitant incidence of anxiety and depression is higher than that of normal people (Koloski et al., 2020; Barberio et al., 2021). This study found that rats in the model group exhibited depressive behaviors, manifested as reduced activity and a decrease in the total distance traveled in the open field test, while WMS improved the depressive behaviors of the model rats to a certain extent. Moreover, the main pathological feature of CG is the chronic inflammatory response of the gastric mucosa (Azer et al., 2025). The long-term presence of inflammation in the gastric mucosal epithelium activates immune cells and continuously releases inflammatory mediators, causing a cascades amplification effect of the inflammatory response. This disrupts the normal physiological structure of gastric mucosal epithelial cells, leads to gastric mucosal injury, and induces an increase in cell proliferation rate, further promoting other pathological changes in the gastric mucosa (Yoshikawa and Naito, 2000). Pharmacological studies have shown that the ingredients in WMS, such as Bupleurum (Teng et al., 2023), Astragalus (Klichkhanov and Suleimanova, 2024), Aurantii fructus (Lan et al., 2023), Angelica dahurica (Zhao et al., 2022), Bletilla striata (He et al., 2017), Frankincense (Efferth and Oesch, 2022), and Myrrh (Batiha et al., 2023), all have anti-inflammatory effects, which reduced the release or generation of inflammatory mediators and may play a good anti-inflammatory role in CG. This study also found that the intrinsic glandular arrangement of the gastric mucosa in the model rats was disordered, and inflammatory cells infiltrated, and the expression levels of pro-inflammatory cytokines in the gastric tissue were all increased. WMS could significantly improve the pathological morphology of gastric tissues and reduce the levels of pro-inflammatory cytokines in model rats, suggesting that WMS may protect the gastric mucosa of CG rats by exerting anti-inflammatory effects. In addition, WMS significantly promoted the expression of gastric mucosal barrier proteins. It is well known that the gastric mucosal barrier is an important mechanism for protecting gastric tissue from acidic environments and erosion by digestive enzymes (Meyer and Goldenring, 2018). In CG, multiple factors can weaken the barrier function, leading to barrier damage that causes hydrogen ions in the gastric lumen to diffuse backward into the gastric mucosa, triggering local inflammatory responses and cellular damage (Xiao, 1983).Our research confirmed that WMS exerted a protective effect on CG and LSDCG, manifested as alleviating the inflammatory response, improving mucosal injury, and mucosal barrier dysfunction. And WMS also significantly regulated the intestinal microbiota and BAs in model rats.

The intestine is a central component of human digestive system and the largest immune organ in the body (Roh et al., 2021). It is home to hundreds of millions of microorganisms, which are closely related to the physiological and pathological activities (Shalon et al., 2023). Once the structure and content of this microorganism change, the overall intestinal microbiota will also be altered, which causes various gastrointestinal and physical diseases (Fu et al., 2022). Existing researches have shown that the abundance and diversity of gastric microbial species in chronic superficial gastritis patients and intestinal metaplasia have changed (Liu et al., 2022). Huangqi Jianzhong Tang can treat chronic atrophic gastritis by changing the gut microbiota (Zhang et al., 2023). Cinnamomi Cortex can also exert its therapeutic effect on chronic atrophic gastritis through the metabolic pathway of the intestinal microbiota (Liu Y. et al., 2023). Numerous studies suggest that targeted regulation of the gut microbiota may be an effective way to treat CG (Sgambato et al., 2017). This research also disclosed that the gut microbiota is disordered in the CG and LSDCG models, and WMS can significantly control the composition of the intestinal microbiota in the CG and LSDCG rats. The dominant microbiota involved *Cryptobacteroides*, *Acutalibacter*, unclassified_Muribaculaceae, *Ligilactobacillus*, *Paramuribaculum*, *Duncaniella*, *Paraprevotella*, *Limosilactobacillus*, CAG-485, *Dysosmobacter*, CAG-873, and *Mailhella*, etc. It is worth noting that the intestinal microbiota is involved in regulating the metabolic process of BAs (Cai et al., 2022b). Studies show that an imbalance in the gut microbiota can lead to disorders in BA metabolism, which in turn can cause intestinal barrier dysfunction and chronic inflammation (Jia et al., 2018). Similarly, BAs can also disturb the composition of the gut microbiota. Deoxycholic acid has been proven to disrupt the gastric microbiome during the transition from CG to intestinal metaplasia (Jin et al., 2022). BAs are important substances synthesized by the liver, and form the “gut-liver axis” between the intestine and the liver (Simbrunner et al., 2021). The physiological functions of the gastrointestinal tract can be affected by regulating the synthesis, metabolism, and reabsorption of BAs (van de Peppel et al., 2019). This study showed that WMS significantly regulated BA metabolism in the intestine and serum of model rats. The main differential BAs include THDCA + TUDCA, CDCA, T-α-MCA, DCA, NorDCA, etc. THDCA is a conjugated BA, which has intestinal anti-inflammatory effects and can alleviate ulcerative colitis (Lv et al., 2023). Similarly, TUDCA can inhibit NF-kB signal transduction and alleviate gastritis caused (Kim et al., 2022). CDCA is a secondary BA that can induce the expression of CDX2 related to gastrointestinal metaplasia (Zhou et al., 2018). DCA is the secondary BA with the highest content in the gastric juice of gastric cancer, and its level increases sequentially with chronic superficial gastritis, atrophic intestinal metaplasia, and the progression of gastric cancer (Lee et al., 2020). NorDCA is also a pro-inflammatory BA derivative, and its acute administration can induce liver inflammation and injury, while disrupting the integrity of the intestinal barrier (Guo et al., 2024). This study suggested that WMS may play an important role in CG by regulating the expression of various BAs. Notably, the differential BAs in the colonic contents and serum are both enriched in the pathways of “Bile secretion” and “Primary bile acid biosynthesis”. Existing studies have confirmed that PPARα and RXR play core regulatory roles in these two metabolic pathways. On the one hand, PPARα promotes the biosynthesis of primary BAs by regulating the levels of key enzymes such as CYP7A1, and simultaneously regulates the levels of BA transporters to enhance bile secretion (Li and Chiang, 2009; Xie et al., 2019). On the other hand, RXR dynamically regulates the expression of related synthetic genes and promotes BA secretion by sensing BA levels (Chiang, 2004). Our research further showed that WMS significantly promoted the levels of PPARα and RXR. To identify which components of WMS might contribute to this effect, a target-directed network pharmacology analysis was performed. Active constituents of the 11 botanical drugs in WMS were retrieved from the TCMSP database and screened for targets annotated as PPARα and RXRα. The resulting drug–component–target network is presented in Supplementary Figure 2. Several candidate components were predicted to interact with PPARα/RXRα, including flavonoids (e.g., quercetin, naringenin, formononetin, calycosin), fatty acids (e.g., oleic acid, linoleic acid), and terpenoids (e.g., β-caryophyllene, farnesol, β-elemene) from *Bupleuri Radix*, *Aurantii Fructus*, *Astragali Radix*, and other botanical drugs in WMS. However, these predictions are based on literature-documented constituents and require experimental validation. In addition, the number of gastric Lgr5+ cells in the model group rats was significantly decreased, and WMS significantly promoted their expression.

Lgr5+ stem cells are a key cell type for gastric epithelial regeneration, exhibiting both self-renewal capacity and multipotent differentiation potential toward gastric epithelial cell types, and are crucial for repairing the damaged gastric mucosal barrier (Ray, 2017). Studies have shown that Jian-Pi-Yi-Qi-Fang improves the repair ability of the gastric mucosal barrier by promoting the proliferative capacity and differentiation potential of Lgr5+ stem cells (Wang et al., 2022). *Helicobacter pylori* increases the function of Lgr5+ stem cells by stimulating the NF-κB signaling (Nascakova et al., 2024). In gastritis diseases, promoting the rapid proliferation and differentiation of stem cells into the mucosal epithelial cell lineage is a promising therapeutic target, especially Lgr5+ gastric stem cells (Liu M. et al., 2023). Furthermore, studies have shown that BAs can regulate the differentiation of gastric Lgr5+ stem cells by activating FXR and TGR5 receptors, thereby inducing intestinal metaplasia (Fu et al., 2019; Sorrentino et al., 2020). This study showed that the number of Lgr5+ cells in the gastric tissues of CG and LSDCG model rats was significantly reduced, WMS significantly promoted the number of Lgr5+ cells in model rats and promoted the levels of cell markers such as Muc5ac, H + -K + ATPase, Pgc, Muc6, and Sst. It is suggested that that WMS treatment is associated with enhanced Lgr5^+^ cell expression and differentiation, such as surface mucous cells, parietal cells, chief cells, mucous neck cells, and endocrine cells. And immunofluorescence co-localization analysis showed that WMS significantly increased the expressions of PPARα, CPT1A, and VLCAD in Lgr5+ gastric stem cells, supporting a spatial link between PPARα/FAO signaling and the Lgr5+ stem cell compartment. BA receptor PPARα, as a nuclear hormone receptor and also an important metabolic regulatory factor, can promote fatty acid oxidation (FAO) by activating specific genes and improve the energy metabolism efficiency of cells (Tahri-Joutey et al., 2021). CPT1A, and VLCAD are also key genes in FAO (Adeva-Andany et al., 2019; Liang, 2023). Existing studies have reported that FAO is necessary for the renewal of Lgr5+ stem cells (Chen et al., 2020). This study found that WMS treatment was associated with elevated levels of PPARα and FAO-related genes, suggesting a potential link between BA metabolism, PPARα signaling, and the regulation of Lgr5+ cell regeneration and differentiation. While previous studies have independently linked BAs to stem cell regulation and FAO to stem cell renewal, the present study extends these findings by showing that WMS treatment is associated with coordinated changes in gut microbiota, BA metabolism, and PPARα/FAO signaling in Lgr5+ gastric stem cells in the context of LSDCG.

Several limitations should be acknowledged. First, the study does not provide direct causal evidence that WMS treatment causes the upregulation of PPARα, CPT1A, VLCAD, and FAO-related genes in Lgr5+ gastric stem cells. The observed changes are based on group comparisons, and causal validation (e.g., PPARα inhibition or knockdown, WMS withdrawal, or dose–response/time-course experiments) was not performed. As a result, the relationship between WMS treatment and these gene expression changes should be interpreted as an association rather than a demonstrated causal effect. Second, the sample size (n = 6 per group) may limit statistical power, particularly for multi-omics analyses, although the consistency of the observed effects across multiple endpoints provides some reassurance. Third, the administered doses exceed the threshold of the recommended daily extract dose of 1 g/kg recommended by best-practice guidelines, and the possibility of non-specific effects cannot be excluded. Fourth, the candidate components predicted to interact with PPARα/RXRα were identified through network pharmacology based on literature-documented constituents. These predictions were not experimentally validated, and the presence and concentrations of these components in the administered WMS preparation remain unverified. Therefore, the network pharmacology results should be considered hypothesis-generating rather than confirmatory. Future studies with larger cohorts, causal validation designs, full chemical characterisation of the WMS preparation, and dose optimisation are needed to confirm the robustness and causality of our findings.

## 5 Conclusion

In conclusion, WMS may promote the differentiation and FAO of Lgr5^+^ gastric stem cells, potentially involving the gut microbiota-hepatic BA metabolism axis, ultimately inhibiting inflammatory responses, enhancing gastrointestinal barrier function, and improving gastric mucosal injury to exert therapeutic effects in LSDCG.

## Data Availability

The data presented in the study are deposited in the NCBI Sequence Read Archive (SRA) repository, accession number PRJNA1548860.
