# Supplementary material for: Wei-Mi-Shu Decoction ameliorates gastric mucosal injury in chronic gastritis with liver-stomach disharmony syndrome through the gut microbiota-hepatic bile acid metabolism axis
Source: Front Pharmacol. 2026 Oct 30;17:1983808. doi: 10.3389/fphar.2026.1983808 (PMC13760025; doi:10.3389/fphar.2026.1983808)
Supplement: Supplementary file 1 [file DataSheet1.pdf]

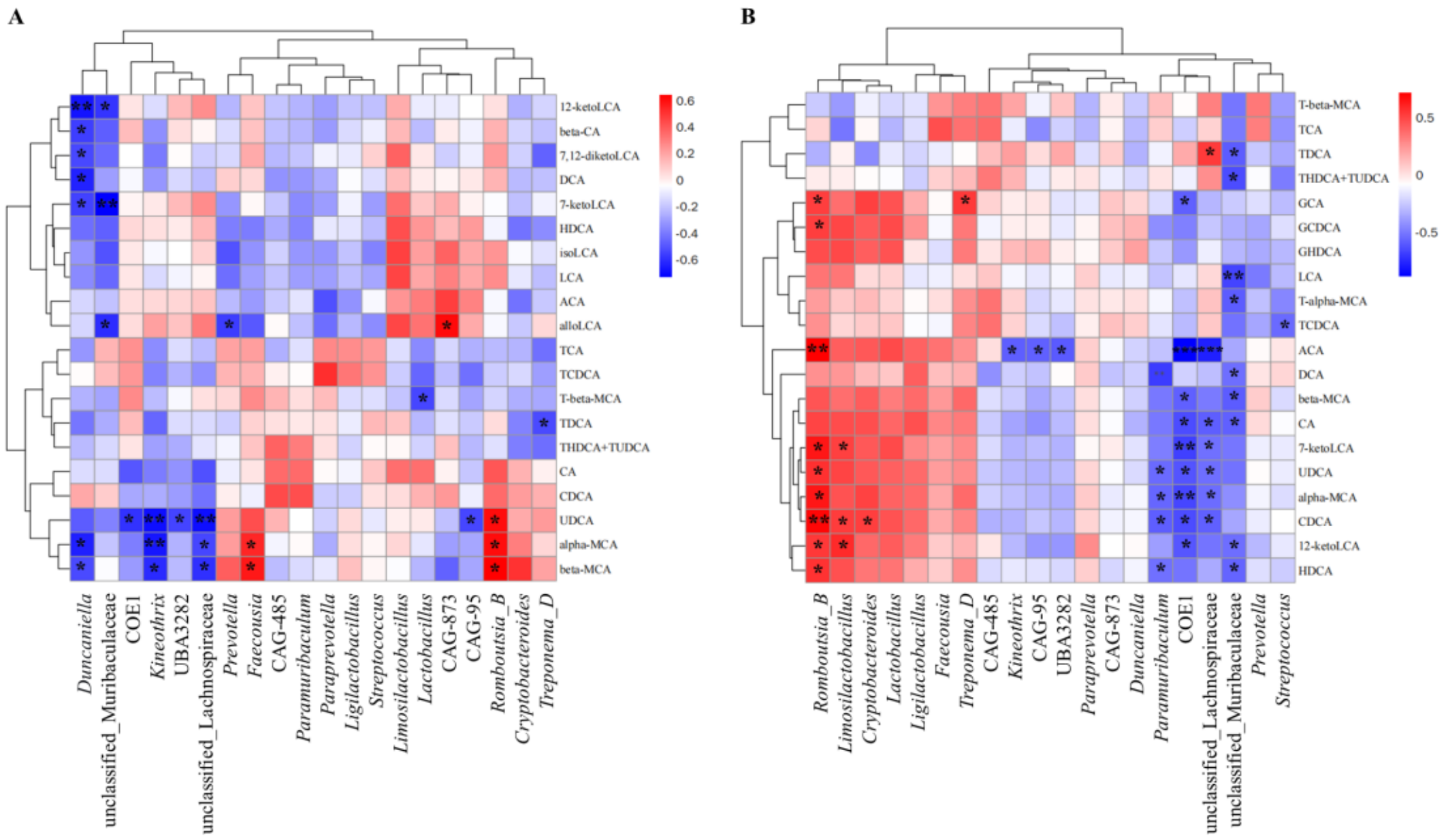

Supplementary Figure 1. Spearman correlation analysis between differential microbial genera and BAs. (A) Correlation heatmap between colonic microbiota and BAs in colonic contents. (B) Correlation heatmap between colonic microbiota and BAs in serum. $^{*}P < 0.05$, $^{**}P < 0.01$, $^{***}P < 0.001$.

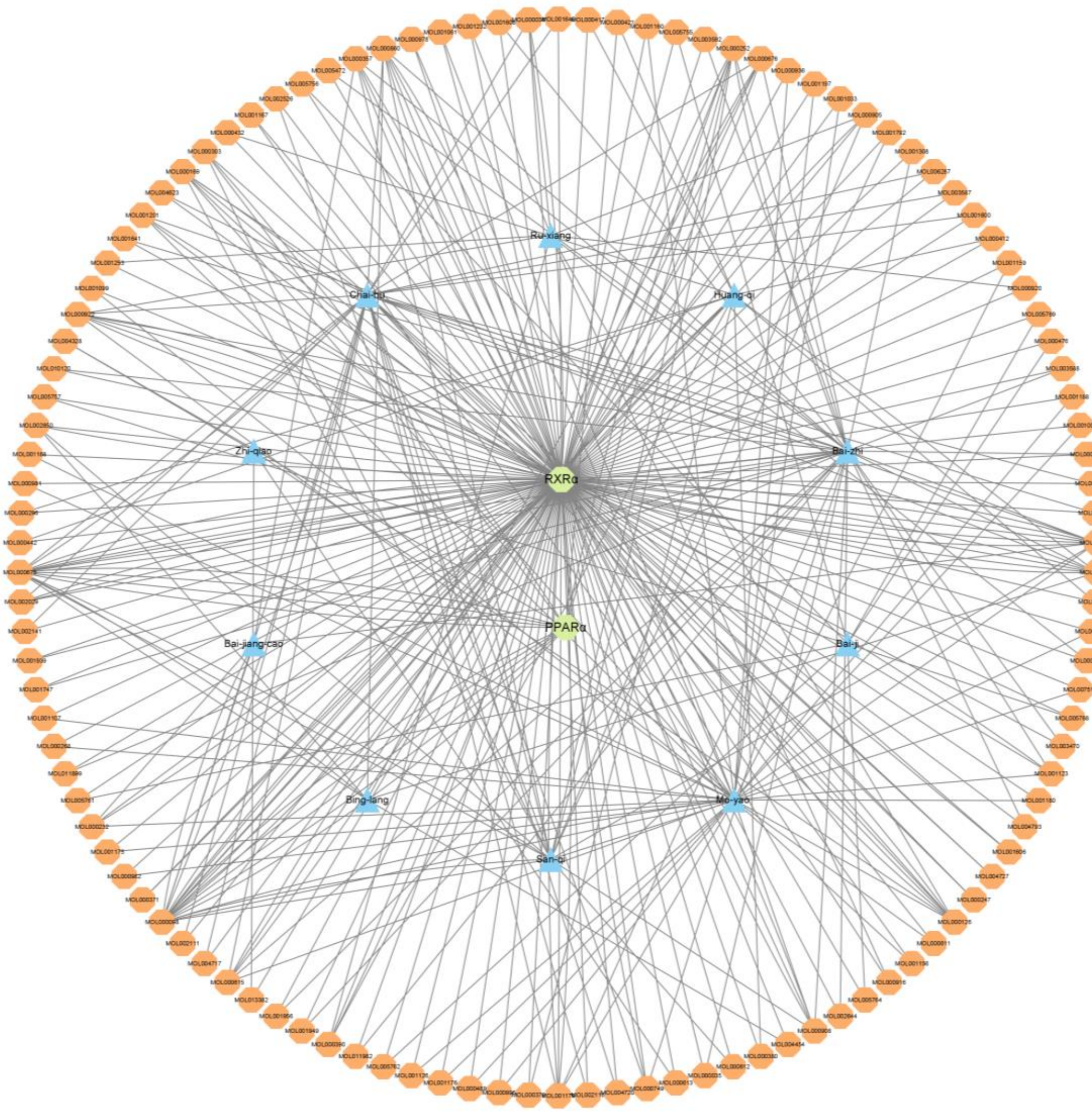

Supplementary Figure 2. Drug–component–target network of WMS predicted to act on the PPARα/RXRα pathway. Active constituents of the 11 botanical drugs in WMS were retrieved from the TCMSP database and screened for targets annotated as PPARα and RXRα. Nodes represent botanical drugs, chemical components, and targets; edges represent drug–component or component–target relationships. Nodes are color-coded by type: botanical drugs (blue), chemical components (orange), and targets (green). The network was constructed using Cytoscape (version 3.9.1).

Supplementary Table 1. Composition and taxonomic information of the 11 botanical drugs in WMS.

No	Chinese name	Pharmacopoeial name	Species name (authority)	Used part	Family
1	Chai-hu	*Bupleuri Radix*	*Bupleurum chinense* DC.	Radix	Umbelliferae
2	Zhi-qiao	*Aurantii Fructus*	*Citrus aurantium* L.	Fructus	Rutaceae
3	Bai-zhi	*Angelicae Dahuricae Radix*	*Angelica dahurica* (Hoffm.) Benth. & Hook.f. ex Franch. & Sav.	Radix	Umbelliferae
4	Bai-ji	*Bletillae Rhizoma*	*Bletilla striata* (Thunb.) Rchb.f.	Rhizome	Orchidaceae
5	San-qi	*Notoginaeng Radix ET Rhizoma*	*Panax notoginseng* (Burkill) F.H.Chen	Radix and Rhizome	Araliaceae
6	Ru-xiang	*Olibanum*	*Boswellia sacra* Flueck.	Resin	Burseraceae
7	Mo-yao	*Myrrha*	*Commiphora myrrha* (Nees) Engl.	Resin	Burseraceae
8	Wa-leng-zi	*Arcae Concha*	*Scapharca broughtonii* (Schrenck)	Concha	Arcidae
9	Huang-qi	*Aatragali Radix*	*Astragalus membranaceus* (Fisch.) Bunge	Radix	Leguminosae
10	Bing-lang	*Arecae Semen*	*Areca catechu* L.	Fructus	Palmae
11	Bai-jiang-cao	*Herba Patriniae*	*Patrinia scabiosifolia* Fisch. ex Trevir.	Herba	Valerianaceae

Notes: WMS, Wei-Mi-Shu Decoction.
